# Phytochemical Analysis of *Agrimonia pilosa* Ledeb, Its Antioxidant Activity and Aldose Reductase Inhibitory Potential

**DOI:** 10.3390/ijms18020379

**Published:** 2017-02-10

**Authors:** Set Byeol Kim, Seung Hwan Hwang, Hong-Won Suh, Soon Sung Lim

**Affiliations:** 1 Department of Food Science and Nutrition, Hallym University, 1 Hallymdaehak-gil, Chuncheon, Gangwon-do 24252, Korea; jwsbcb0187@naver.com (S.B.K.); isohsh@gmail.com (S.H.H.); 2 Institute of Natural Medicine, College of Medicine, Hallym University, 1 Hallymdaehak-gil, Chuncheon, Gangwon-do 24252, Korea; hwsuh@hallym.ac.kr

**Keywords:** *Agrimonia pilosa* Ledeb, aldose reductase, flavonoids, 1,1-diphenyl-2-picrylhydrazyl (DPPH), diabetic complication

## Abstract

The aim of this study was to determine aldose reductase (AR) inhibitory activity and 1,1-diphenyl-2-picrylhydrazyl (DPPH) free radical scavenging activity of compounds from *Agrimonia pilosa* Ledeb (AP). We isolated agrimoniin (AM), four flavonoid glucosides and two flavonoid glucuronides from the *n*-butanol fraction of AP 50% methanol extract. In addition to isolated compounds, the AR-inhibitory activity and the DPPH free radical scavenging activity of catechin, 5-flavonoids, and 4-flavonoid glucosides (known components of AP) against rat lens AR (RLAR) and DPPH assay were measured. AM showed IC_50_ values of 1.6 and 13.0 μM against RLAR and DPPH scavenging activity, respectively. Additionally, AM, luteolin-7-*O*-glucuronide (LGN), quercitrin (QU), luteolin (LT) and afzelin (AZ) showed high inhibitory activity against AR and were first observed to decrease sorbitol accumulation in the rat lens under high-sorbitol conditions ex vivo with inhibitory values of 47.6%, 91.8%, 76.9%, 91.8% and 93.2%, respectively. Inhibition of recombinant human AR by AM, LGN and AZ exhibited a noncompetitive inhibition pattern. Based on our results, AP and its constituents may play partial roles in RLAR and oxidative radical inhibition. Our results suggest that AM, LGN, QU, LT and AZ may potentially be used as natural drugs for treating diabetic complications.

## 1. Introduction

Aldose reductase (AR, EC.1.1.1.21) is a key enzyme in the polyol pathway that catalyzes the conversion of glucose to sorbitol in the hyperglycemic state and oxidoreductase-induced nicotinamide adenine dinucleotide phosphate (NADPH) to NADP^+^ [1]. Accumulation of sorbitol leads to the generation of osmotic stress, an influx of water and causes of diabetic complications such as cataracts and retinopathy. In addition, the conversion of sorbitol to fructose is catalyzed by nicotinamide adenine dinucleotide (NADH)-dependent sorbitol dehydrogenase. Increased fructose formation leads to the formation of reactive dicarbonyl species such as glucosones, glyoxal, and methylglyoxal, which are important factors in advanced glycation end products [2]. Oxidative stress causes an imbalance between the formation of free radicals and the body’s antioxidant potential. Free radicals are defined as atoms or molecules that contain one or more unpaired electrons [3]. Diabetes mellitus and its complications, such as retinopathy, nephropathy, neuropathy, and atherosclerosis, are caused by an imbalance in cells and free radicals, and this imbalance is mainly responsible for the auto-oxidation of glucose and glycosylated proteins [4,5] Therefore, the development of diabetic complications could be controlled by inhibiting AR activity and also by increasing antioxidant activity in the body.

*Agrimonia pilosa* Ledeb (*A. pilosa*, AP), belonging to the Rosaceae family, is famous in traditional Chinese medicine. According to pharmacological studies, it has anti-nociceptive, anti-inflammatory, antioxidant, anticancer and α-glucosidase inhibitory activity [6,7]. The known constituents of AP are 3-methoxy quercetin, quercitrin (QU), quercetin (QC), tiliroside, ursolic acid, tormentic acid and corosolic acid [8,9]. The major known flavonoids of AP are catechin (CT), luteolin (LT), QC, isoquercetin (IQC), hyperin (HP), apigenin (AG), vitexin (VT), kaempferol (KP), astragalin (AS), and afzelin (AZ) [10,11,12]. Generally, these flavonoids are involved in plant metabolism and possess antioxidant, antidiabetic, anticancer, and various inhibitory activities [13,14].

The isolation and purification of active compounds from complex plant extracts takes a long time. In the past few years, several online high performance liquid chromatography (HPLC) methods that use post-column derivative method, which are based on online detection by 1,1-diphenyl-2-picrylhydrazyl (DPPH) or 2,2′-azino-bis(3-ethylbenzothiazoline-6-sulphonic acid) (ABTS) radicals, have been utilized to screen antioxidants in some complex plant extracts to avoid the aforementioned problem. These methods required an online instrumental system and technical skills that were complex and available. Recently, the more convenient offline DPPH-HPLC method was successfully developed by spiking the complex plant extracts [15,16].

To date, no data have been published on the inhibitory effects of AP on rat lens AR (RLAR), DPPH radical scavenging capacity and offline DPPH-HPLC assay. Therefore, the inhibitory effects of ten known flavonoids from the literature as well as seven compounds isolated from AP 50% methanol (MeOH) extract (APE) were investigated to evaluate their use in treatment of RLAR-related diabetic complications. The active compounds of APE that showed antioxidant properties were investigated by offline DPPH-HPLC assay. Additionally, the ability of the major active compounds to decrease sorbitol accumulation in rat lens in ex vivo high-sorbitol conditions as well as the recombinant human AR (RHAR) inhibition type of the compounds were assessed.

## 2. Results

### 2.1. Structural Determination of Isolated Compounds

The effects of APE on RLAR and DPPH free radical scavenging activity were further investigated. APE exhibited inhibitory activity against RLAR, with 51.4% inhibition at a concentration of 10.0 μg/mL. Moreover, APE showed 53.4% inhibition of DPPH at a concentration of 7.1 μg/mL. Consequently, APE was further partitioned by systematic fractionation. Among the resulting fractions, the ethyl acetate (EtOAc) and *n*-butanol (*n*-BuOH)-soluble fractions exhibited potent inhibitory activity against RLAR with 84.4% and 92.4% inhibition, respectively, compared with the positive control tetramethylene glutaric acid (TMG; 99.7% inhibition) at a concentration of 1.0 μg/mL. The EtOAc and *n-*BuOH fractions also showed inhibitory activity against DPPH, with 62.3% and 61.0% inhibition, respectively, compared with the positive control l-ascorbic acid (81.0% inhibition) (Table 1).

**Figure 1:**
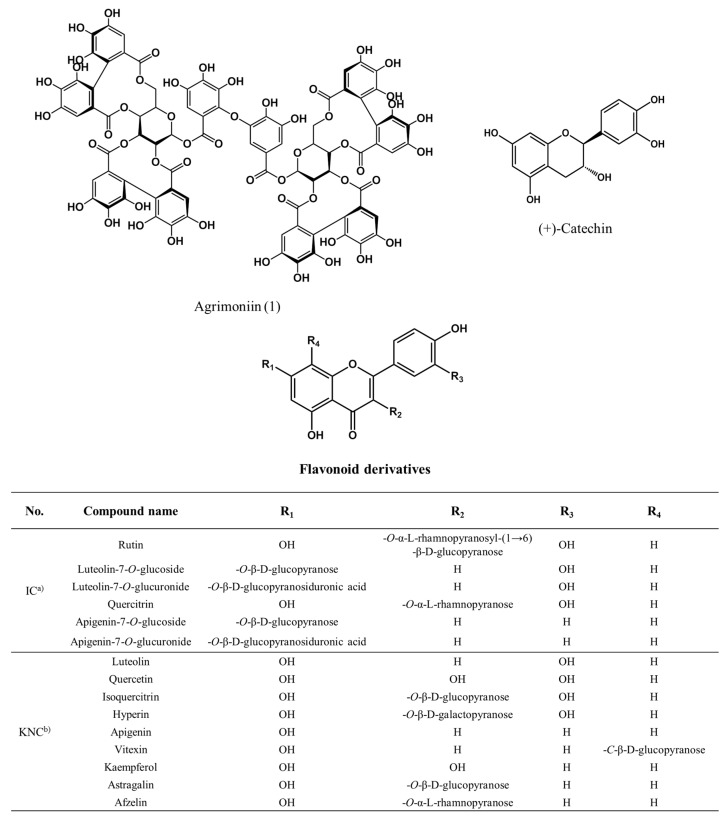
The structure of the compounds known and isolated from the *n*-BuOH fraction of *A. pilosa* Ledeb; ^a)^ IC is the compounds isolated from *A. pilosa* Ledeb; ^b)^ KNC is the known compounds isolated from *A. pilosa* Ledeb.

**Figure 2:**
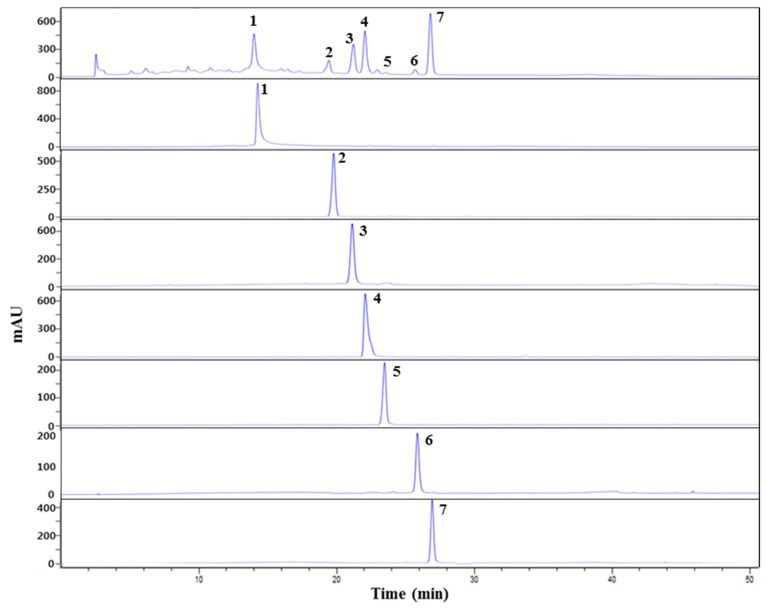
HPLC chromatogram of the compounds isolated from the *n*-BuOH fraction of *A. pilosa* Ledeb. at 254 nm; Peak 1: agrimoniin; Peak 2: rutin; Peak 3: luteolin-7-*O*-glucoside; Peak 4: luteolin-7-*O*-glucuronide; Peak 5: quercitrin; Peak 6: apigenin-7-*O*-glucoside; Peak 7: apigenin-7-*O*-glucuronide.

Therefore, this study focused on the isolation of the AR inhibitor from the *n*-BuOH fraction. The seven compounds isolated from *n*-BuOH were identified as compound 1 (agrimoniin, AM, 69.3 mg) [17], compound 2 (rutin, RT, 30.3 mg) [18], compound 3 (luteolin-7-*O*-glucoside, LGC, 96.1 mg) [19], compound 4 (luteolin-7-*O*-glucuronide, LGN, 150.5 mg) [20], compound 5 (quercitrin, QU, 11.6 mg) [21], compound 6 (apigenin-7-*O*-glucoside, AGC, 21.3 mg) [22] and compound 7 (apigenin-7-*O*-glucuronide, AGN, 251.6 mg) [23] on the basis of the 1D and 2D NMR spectral data (Table S1), as well as by comparison with published spectral data (Figure 1 and Figure 2).

### 2.2. Inhibitory Effect of Isolated Compounds on RLAR

We compared the ability of the isolated compounds and TMG (a positive control) to inhibit RLAR activity (Table 2). Among the isolated constituents, RT, LGC and AGC exhibited RLAR inhibitory activity, with 50% inhibition concentration (IC_50_) values of 9.5, 8.1 and 4.3 μM, respectively. QU had the highest IC_50_ value of 0.2 μM, which was 2.5 times higher than the positive control (IC_50_ of TMG = 0.5 μM). AM and LGN had high IC_50_ values at 1.6 and 0.7 μM, respectively, while AGN was inactive. In addition, previous investigations of flavonoids isolated from AP by Jiang et al., Kato et al., and Liu et al. reported that CT, LT, QC, IQC, HP, AG, VT, KP, AS, and AZ were isolated from the leaves of AP [10,11,12]. The RLAR inhibitory effects of ten known compounds were evaluated using TMC as a positive control. LT and AZ had the strongest RLAR inhibitory activity, with IC_50_ values of 0.6 and 1.0 μM, respectively. QC, IOC, HP, AG and AS also exhibited potent inhibitory activity, with IC_50_ values ranging from 3.2 to 15.2 μM. CT and VT showed lower inhibitory activity, with 7.2% and 12.2% inhibition, respectively, against RLAR at a concentration of 10.0 μg/mL. The RLAR inhibitory effect of ten known compounds was similar to previous data from the literature [24,25].

### 2.3. DPPH and Off-Line DPPH HPLC Assay

**Figure 3:**
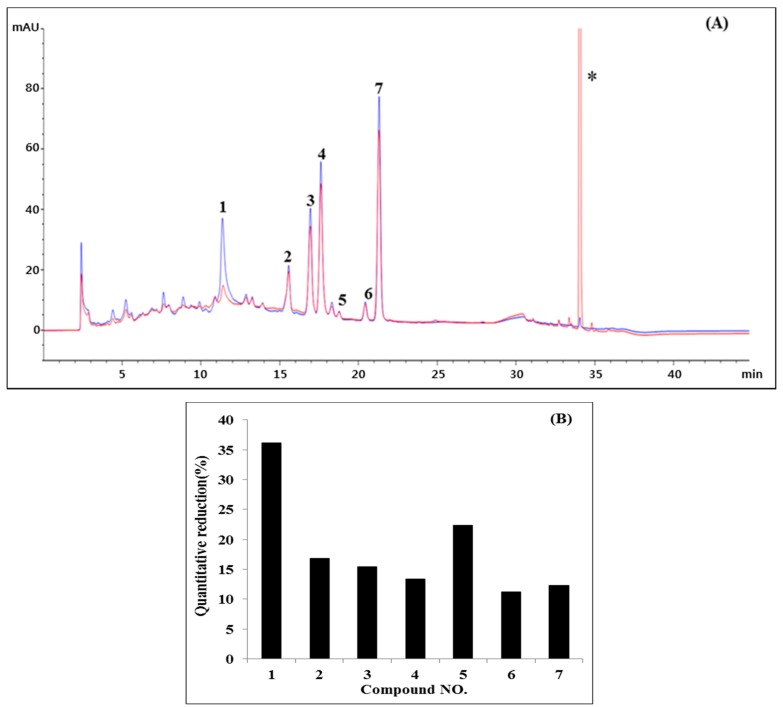
HPLC-ultraviolet (UV) (before reaction: blue) and DPPH-HPLC-UV (after reaction: red) of the *n*-BuOH fraction from *A. pilosa* Ledeb. at 254 nm (**A**) and quantitative reduction (%) in the peak areas of compounds designated as follows (**B**); Peak 1: Agrimoniin; Peak 2: Rutin; Peak 3: Luteolin-7-*O*-glucoside; Peak 4: Luteolin-7-*O*-glucuronide; Peak 5: Quercitrin; Peak 6: Apigenin-7-*O*-glucoside; Peak 7: Apigenin-7-*O*-glucuronide; * is DPPH.

The *n*-BuOH fraction showed high DPPH radical scavenging activity. The chromatogram of the *n*-BuOH fraction without DPPH (blue line) and with DPPH (red line) is shown in Figure 3A, which presents the peak areas of seven compounds isolated reduced obviously. As shown in Figure 3B, seven compounds showed peak area reduction (PAR) between 13.6%–37.4%. In addition, Zeng et al. reported that the rear eluting peak of the 34-min retention time is of DPPH [15]. Among these seven compounds, AM and QU (PAR 23%–37%) would be more potent antioxidants than RT, LGC, LGN, AGC and AGN, which showed PAR lower than 20.0%. The results of seven compounds in *n*-BuOH fraction suggested antioxidant activity. The scavenging activity of the seven compounds isolated from the *n*-BuOH fraction of APE was evaluated by measuring DPPH free radical scavenging activity (Table 3). Of the tested compounds, AM had the highest IC_50_ value at 13.0 μM. RT, LGC, LGN and QU also showed strong scavenging activity with IC_50_ values of 66.8–80.6 μM, compared to the positive control, l-ascorbic acid (IC_50_ = 147.3 μM). However, AGC and AGN had almost no DPPH free radical scavenging activity. Scavenging activity of the known compounds from AP was evaluated using l-ascorbic acid. Of the tested known compounds, LT, QC, IOC, HP, and KP showed strong scavenging activity, with IC_50_ values of 88.2, 70.4, 65.9, 73.3, and 91.6 μM, respectively, which were higher than those of the positive control (l-ascorbic acid = 147.3 μM). On the other hand, VT, AS, and AZ showed no DPPH free radical scavenging activity.

### 2.4. Kinetic-Type RHAR Inhibition by the Active Compounds

**Figure 4:**
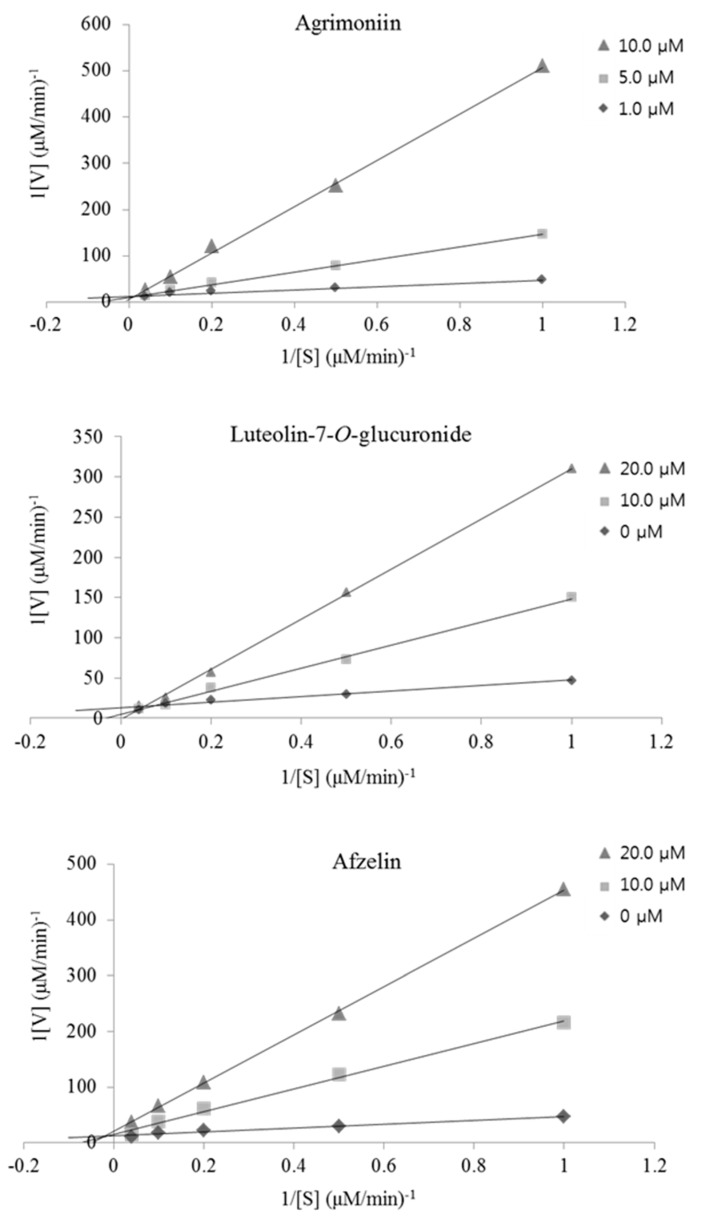
Lineweaver–Burk plots showing the reciprocal of the velocity (1/V) of recombinant rat lens aldose reductase versus the reciprocal of the substrate concentration (1/S) with _DL_-glyceraldehyde as the substrate at concentrations of 1.0 to 25.0 mM.

A kinetic study using _DL_-glyceraldehyde as a substrate at a concentration range of 1.0 to 25.0 mM was performed to determine the type of inhibition that AM, LGN and AZ exhibited, which showed the highest activity. The kinetic analysis of RHAR inhibition shown in Figure 4 was conducted with AM, LGN and AZ using Lineweaver–Burk plots of 1/velocity and 1/concentration of substrate. With the change of the concentration of the substrate dl-glyceraldehyde, the slopes obtained with the uninhibited enzyme and the three different concentrations of each compound were found to be parallel. The results showed that the inhibition of RHAR by AM, LGN and AZ were competitive and mixed-type. In addition, Lee et al., Ha et al., and Chethan et al. reported that QU, LT, and QC showed uncompetitive, mixed-type, and noncompetitive inhibition patterns, respectively, against RHAR (Table 3) [31,32,33].

### 2.5. Lens Culture and Intracellular Sorbitol Measurement

We also investigated the effect of RLAR inhibitory compounds (including the known inhibitory compounds LT and AZ) on the sorbitol accumulation in isolated rat lens; the results are shown in Table 3. AM, LGN, QU, LT and AZ inhibited sorbitol accumulation by 47.6%, 91.8%, 76.9%, 91.8%, and 93.2%, respectively, at a concentration of 5.0 μg/mL. In addition, QC as a positive control, which inhibits sorbitol accumulation in isolated rat lens by 85.7%, reduced the sorbitol level in culture medium containing a high glucose concentration. These results indicated that RLAR inhibitors isolated from APE are effective in either preventing or slowing sugar cataract formation associated with diabetes.

## 3. Discussion

**Figure 5:**
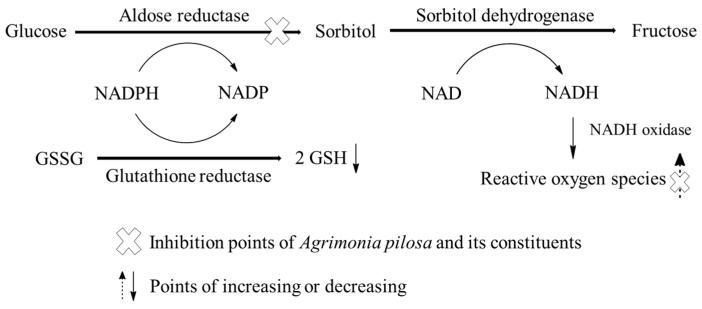
Inhibition points of *A. pilosa* Ledeb and its constituents on polyol pathway. GSH: glutathione, GSSG: glutathione disulfide, NAD: nicotinamide adenine dinucleotide, NADH: oxidoreductase-induced nicotinamide adenine dinucleotide, NADP: nicotinamide adenine dinucleotide phosphate, NADPH: oxidoreductase-induced nicotinamide adenine dinucleotide phosphate.

The results of the RLAR and DPPH revealed that all tested APEs have a potent inhibitory effect on RLAR and protect against oxidative stress (Figure 5); these results are shown in Table 1. In addition, the inhibitory effect of the *n*-BuOH fraction of APE on RLAR inhibition was comparable to that of the positive control TMS. In previous studies, VT, RT, HP, LT-7-*O*-β-d-glucopyranoside, QC, tiliroside, LT, AP, and KP isolated from AP were analyzed by HPLC-UV and showed α-glucosidase inhibitory activity, ABTS^+^ radical scavenging activity, and hydroxyl radical scavenging activity [12]. QC-3-*O*-β-d-glucopyranoside, QC-3-*O*-α-l-rhamnopyranoside, (2*S*,3*S*)-(−)-taxifolin-3-*O*-β-d-glucopyranoside, KP-3-*O*-α-l-rhamnopyranoside, 1-butanoyl-3,5-dimethyl-phloroglucinyl-6-*O*-d-glucopyranoside, CT, tiliroside, AG, and agrimonolide in AP were established for characterization and simultaneous quantification by the HPLC-diode array detector-electrospray ionization mass spectrometry (MS)/MS method [10]. Kato et al successfully separated three new compounds and nine known compounds, including (−)-aromadendrin-3-*O*-β-d-glucopyranoside, desmethylagrimonolide-6-*O*-β-d-glucopyranoside, and 5,7-dihydroxy-2-propylchromone-7-*O*-β-d-glucopyranoside, agrimonolide-6-*O*-glucoside, takanechromone C, AT, AZ, tiliroside, LT, QC, IQC, and AGC from AP's aerial parts MeOH extract [11].

Various flavonoid constituents were isolated as active compounds from AP. Based on the literature, we evaluated the effect of ten known flavonoids and isolated compounds from the *n*-BuOH fraction of APE on RLAR. Among the compounds isolated, compound 4 was isolated for the first time from this plant and AM (IC_50_ = 1.6 μM) was evaluated in RLAR for the first time. Except for AGN, all compounds showed a potent inhibitory effect, with IC_50_ values of 0.2–9.5 μM. Among active compounds, LGN and QU had similar or higher activity than the positive control TMS. Previous flavonoid RLAR studies reported that LGC (7.5 μM) [26], LGN (3.1 μM), LT (0.5 μM) [27], and AGC (23.0 μM) [28] were isolated from plant sources. Matsuda et al. reported potent IC_50_ values as follows: QC (2.2 μM), IQC (4.5 μM), HP (3.0 μM), AG (2.2 μM), KP (10.0 μM), and RT (9.0 μM) [24]. QU had an IC_50_ value of 0.2 μM [29]. These reported data were similar to our experimental data (shown in Table 2). The sorbitol accumulation was not significantly correlated with RLAR activity. AM, LGN, QU, LT, AZ, and QC showed different RLAR inhibitory effects (IC_50_ values) as structures (QU, 0.2 μM > LT, 0.6 μM > LGN, 0.7 μM > AZ, 1.0 μM > AM, 1.6 μM > QC, 3.2 μM). On the other hand, high inhibition of sorbitol accumulation was observed in the following order: AZ (93.2%), LGN (91.8%) and LT (91.8%), QC (85.7%), QU (76.9%), and AM (47.6%). According to structures of flavonoids, different inhibitory effects were seen in vitro and ex vivo. Therefore, this result suggests that bioavailability may be affected by structures of flavonoids.

Based on these results, the RLAR inhibitory effect of flavonoid derivatives and the structure activity relationship (SAR) were investigated using the RLAR assay. RLAR inhibitory effects of flavonoid derivatives depend on the position and sugar type of the aromatic A and C ring at a catechol moiety. RT, QC, IQC, HP, and QU were isomers of flavonol and had rhamnoside, no sugar, galactoside, glucoside, and rutinoside, respectively, in the same position. However, these compounds showed different RLAR inhibitory effects and different IC_50_ values. RT showed IC_50_ values 15.5, 20.0, 25.5, and 47.5 times higher than those of QC, HP, IQC, and QU, respectively. In addition, LT derivatives showed different RLAR inhibitory effects according to sugar types (LGN > LT > LGC). Flavone derivatives showed different patterns on SAR. AZ, AG, AGN, AG, KP, and AGN have no hydroxyl at the 4’ position at a catechol moiety B ring, and showed lower activity than the flavonol structure. However, rhamnoside at the 3-position in the A ring of flavonol/flavone structure showed stronger activity than other sugar types, and glucuronide and glucoside at the 7′ position showed higher activity than glucoside at the 3′ and 7′ positions. A previous SAR study demonstrated that the inhibitory activity of flavonol/flavone was different according to 3′,4′-hydroxyl moiety in a catechol moiety at the B ring, and suggested that sugar type and hydroxyl moieties at the 3’ and 7’ position increased the activity of RLAR [26].

Offline DPPH-HPLC method is able to rapidly screen antioxidants from complex mixtures, especially for natural products with minimum sample preparation. Reduction or disappearance of the peak areas in the HPLC chromatogram certify potential antioxidant activity of the compounds, while there was no change of peak areas for compounds with no antioxidant activity after their reaction with DPPH. Zhang et al. reported that eighteen antioxidants were screened and identified from *Pueraria lobata* flowers by the offline DPPH-HPLC-MS/MS method [34] Moreover, seven antioxidant compounds in *Eucommia ulmoides* Olive were analyzed by offline DPPH-HPLC [35]. As shown in Figure 3, our offline DPPH-HPLC method results suggested that this method is a good strategy for selecting antioxidant compounds from crude plant extracts. Many studies were done for evaluating the antioxidant activities of flavonoids, which showed the ability to quench free radicals through several mechanisms, including the donation of electrons and hydrogen atoms, and chelate transition metals [36]. Thus, we evaluated the antioxidant activity of seven isolated compounds with offline DPPH-HPLC, as well as the DPPH radical scavenging activity of ten known flavonoids. The *n*-BuOH fraction of AP showed the capacity to scavenge DPPH radicals. In addition, AM, RT, LGC, LGN and QU showed potent DPPH inhibitory activity, with IC_50_ values of 13.0, 66.8, 71.5, 80.6, and 77.9 μM, respectively. Among ten known flavonoids, seven compounds (except for VT, AG, and AZ) exhibited potent DPPH inhibitory activity, with IC_50_ values of 65.9–156.3 μM, compared to l-ascorbic acid (147.3 μM, Table 2). Although activity results of offline DPPH-HPLC and DPPH assay showed different activity patterns, we believe that the offline DPPH method can be very efficient and fast for screening antioxidant compounds from complex mixtures (natural products, food, or materials).

## 4. Materials and Methods

### 4.1. General

^1^H and ^13^C NMR spectra and correlation 2D NMR spectra were obtained from a Bruker Avance DPX 400 (or 600) spectrometer. These spectra were obtained at operating frequencies of 400 MHz (^1^H) and 100 (or 150) MHz (^13^C) with CD_3_OD, (CD_3_)_2_SO, (CD_3_)_2_CO, or D_2_O and TMS used as an internal standard; chemical shifts were reported in δ values. Isolated compounds were analyzed by electron ionization-MS in a low resolution-MS equipped with JMS-700. A semi prep-HPLC system for separation identification (recycling preparative LC908-C60, JAI, Tokyo, Japan) was used.

### 4.2. Chemicals and Reagents

l-Ascorbic acid, DPPH, NADPH, DL-glyceraldehyde dimer, TMG, glucose, and the reference compounds used in this study (CT, LT, QC, IQC, HP, AG, VT, KP, AS, and AZ) were purchased from Sigma-Aldrich (St. Louis, MO, USA). RHAR was purchased from Wako Pure Chemical Industries (Osaka, Japan). All other chemicals and reagents used were of analytical grade.

### 4.3. Plant Materials

Dried bark of AP was purchased at a local market in Yeongcheon, Gyeongsangbuk-do Province, Korea (June 2015). The AP was identified by Hyung Joon Chi at Seoul National University, and a voucher specimen (No. RIC-2015-0615) was deposited at the Regional Innovation Center, Hallym University, Korea.

### 4.4. Extraction, Fractionation, and Isolation

The dried bark of AP (10 kg) was extracted twice with methylene chloride (50.0 L × 2 times) for 48 h at room temperature. The suspension was filtered and evaporated under reduced pressure at 40 °C to give methylene chloride extract (yield: 1.9%, 194.0 g). The residue was extracted with APE. The suspension was filtered and evaporated under reduced pressure at 40 °C to give the APE (yield: 7.6%, 762.9 g). This extract was suspended in distilled water and then successively partitioned with EtOAc, *n*-BuOH and water to yield EtOAc (17.2%, 110.0 g), *n*-BuOH (20.6%, 132.0 g) and water fractions (59.5%, 381.3 g), respectively. These fractions were concentrated to dryness by rotary evaporation at 40 °C, while the water fraction was freeze-dried. The *n*-BuOH fraction showed strong inhibitory activity against RLAR. Thus, the *n*-BuOH fraction was applied to an open glass column packed with Diaion HP-20 with MeOH-H_2_O in a gradient of 30%–100% MeOH, thereby yielding 15 sub-fractions (HP-S1 to HP-S15). It was then eluted with water to wash any sugars or impure components. Fraction HP-S8 (200.0 mg) was purified to yield compound 1 (69.3 mg) and compound 2 (30.3 mg) by recycle HPLC with a gradient system from 30%–35% MeOH. Other fractions obtained from Diaion HP-20 were applied to a Sephadex LH-20 column (90 cm × 3 cm, internal diameter). Fraction HP-S9 (888.6 mg) was separated with 70% MeOH to obtain compound 3 (96.1 mg) and compound 4 (150.5 mg). Fraction HP-S11 (797.1 mg) was separated with a 60% MeOH system to obtain compound 5 (11.6 mg) and compound 6 (21.3 mg). Fraction HP-S12 (465.9 mg) was isolated with a 100% MeOH system to yield compound 7 (251.6 mg).

### 4.5. Preparation of Aldose Reductase

Crude RLAR was prepared as follows: lenses weighing 250–280 g were removed from Sprague–Dawley rats and frozen at −70 °C until use. This was approved by the University of Hallym Animal Care and Use Committee (registration number: Hallym 2015-06-08). Non-cataractous transparent lenses were pooled and a homogenate was prepared in 0.1 M phosphate-buffered saline (pH 6.2). RLAR homogenate was then centrifuged at 10,000*× g* for 20 min at 4 °C in a refrigerated centrifuge. The supernatant was collected and used as the RLAR. All procedures were carried out at 4 °C [37].

### 4.6. Determination of RLAR Inhibition In Vitro

RLAR activity was assayed spectrophotometrically by measuring the decrease in the absorption of NADPH at 340 nm over a 3-min period using _DL_-glyceraldehyde as the substrate. Each 1.0 mL cuvette contained equal units of the enzyme, 0.10 M potassium phosphate buffer (pH 6.2), 1.6 mM NADPH, 25 mM _DL_-glyceraldehyde (the substrate), and an inhibitor or dimethyl sulfoxide (DMSO). The inhibition of RLAR (%) was calculated with the following equation: [1 − (∆A sample/min) − (∆A blank/min)/(∆A control/min) − (∆A blank/min)] × 100%, where ∆A sample/min is the reduction of absorbance for 3 min with reaction solution, the test sample, and substrate, and ∆A control/min is the same but with DMSO instead of the test sample [38].

### 4.7. HPLC Analysis

The sample was analyzed using an Agilent Technologies modular model 1200 system with a vacuum degasser (G1322A), a quaternary pump (G1311A), an auto-sampler (G1329A), a thermo-statted column compartment (G1316A), and a variable wavelength detector (VWD, G1314D) system. The separation was achieved on an Eclipse XDB-phenyl column (150 mm × 4.6 mm, 3.5 μm) maintained at 30 °C. The elution solvents were 0.1% trifluoroacetic acid (A) and MeOH (B) with the following gradient: 20%–30% B (0–3 min), 30%–40% B (3–10 min), 40%–50% B (10–20 min), 50%–60% B (25–35 min), 60%–100% B (25–35 min), 100%–100% B (35–38 min), 100%–20% B (38–40 min), and 20%–20% B (40–45 min). Injection volume was 10 µL (sample concentration: 1 mg/mL) and UV wavelength was 254 nm.

### 4.8. Evaluation of DPPH Radical Scavenging Capacity

The stable free radical was used to determine the free radical-scavenging activity of the extracts [39]. Briefly, a 0.32 mM solution of DPPH in MeOH was prepared, and 180 µL of this solution was mixed with 30 µL of each sample at concentrations of 0.05–1.0 mg/mL in DMSO. After 20 min of incubation in the darkroom, the decrease in the absorbance of the solution was measured at 570 nm on a microplate reader (EL800 Universal Microplate reader, Bio-Tek instruments, Winooski, VT, USA). DPPH inhibitory activity was expressed as the percentage inhibition (%) of DPPH in the aforementioned assay system, and was calculated as [1 − (A_sample_ − A_blank_/A_control_ − A_blank_)] × 100%, where A_control_ is the absorbance of DPPH solution (180 µL) with methanol (30 µL); A_blank_ is the absorbance of distilled water (180 µL) with methanol (30 µL); A_sample_ is the absorbance of DPPH solution (180 µL) with sample solution (30 µL).

### 4.9. OffLine DPPH HPLC Assay

The offline DPPH HPLC assay was performed by modifying a previously-described protocol [40]. Thirty microliters (20 mg/mL in MeOH) of the *n*-BuOH fraction from APE were mixed with 180 μL prepared DPPH solution (0.32 mM). The mixture was incubated in the dark for 20 min, then filtered through a 0.45-μm filter for HPLC analysis. The *n*-BuOH (20 mg/mL in MeOH) was used as a control. The extent of peak decrease is expressed as a quantitative reduction.

### 4.10. Determination of Inhibition-Type of RHAR by Active Compound

Reaction mixtures consisted of 0.1 M potassium phosphate, 1.6 mM NADPH, and 2 mM of RHAR with varied concentrations of substrate _DL_-glyceraldehyde and AR inhibitor in a total volume of 600 μL. Concentrations ranged from 0 to 25 mM for DL-glyceraldehyde and from 0 to 20 μM for the active compound. RHAR activity was assayed by measuring the decrease in absorption of NADPH after substrate addition at 340 nm using a Bio Tek Power Wave XS spectrophotometer (Bio Tek Instruments, Winooski, VT, USA) [41].

### 4.11. Lens Culture and Intracellular Sorbitol Measurement

Lenses isolated from 10-week-old Sprague–Dawley rats using the registration number mentioned in the section of 4.5 were cultured for 6 d in TC-199 medium that contained 15% fetal bovine serum, 100 units/mL penicillin, and 0.1 mg/mL streptomycin, under sterile conditions and an atmosphere of 5% CO_2_ and 95% air at 37 °C. Samples were dissolved in DMSO. The lenses were divided into three groups and cultured in medium containing 30 mM glucose and RLAR-active compounds. Each lens was placed in a well containing 2.0 mL of medium. Sorbitol was determined by HPLC using the methods mentioned in the section of 4.7 after its derivatization by reaction with benzoic acid to a fluorescent compound [42].

### 4.12. Statistical Analysis

Inhibition rates were calculated as percentages (%) with respect to the control value, and the IC_50_ value was defined as the concentration at which 50% inhibition occurred. Data are expressed as mean values ± standard deviation of triplicate experiments.

## 5. Conclusions

In summary, seven compounds isolated from the *n*-BuOH fraction of the APE and ten flavonoids known as ingredients of AP were evaluated for RLAR inhibitory activity and DPPH radical scavenging activity. Additionally, antioxidant compounds in the *n*-BuOH fraction of APE were investigated with a DPPH offline HPLC assay. Of the compounds tested, AM, LGN, QU, LT, and AZ showed strong inhibitory activity against RLAR and sorbitol accumulation. Consequently, we conclude that APE and its constituents may play partial roles in RLAR and oxidative radical inhibition. Our results suggest that AP may potentially be used as a herbal drug to treat diabetic complications.

## Figures and Tables

**Table 1:** Inhibitory effect of 50% MeOH extract of *Agrimonia pilosa* Ledeb. on rat lens aldose reductase (RLAR) and 1,1-diphenyl-2-picrylhydrazyl (DPPH) free radical scavenging activity. TMG: tetramethylene glutaric acid.

Extract and Fractions		Inhibition (%)	
		RLAR	DPPH
Methylene chloride extract		6.8 ± 0.20	4.19 ± 0.14
50% MeOH ext.	Crude extract	51.4 ± 0.10	53.4 ± 0.14
	EtOAc fraction	84.4 ± 0.27	62.3 ± 0.04
	*n*-BuOH fraction	92.4 ± 0.14	61.0 ± 0.42
	Water fraction	37.9 ± 0.47	33.0 ± 0.10
RLAR	TMG	99.7 ± 0.11	-
DPPH	Ascorbic acid	-	81.0 ± 0.01

**Table 2:** Inhibitory effect of compounds referenced and isolated from *A. pilosa* Ledeb. on rat lens aldose reductase (RLAR) and DPPH free radical scavenging activity.

Entry	Compounds		DPPH		RLAR	
			Experiments			References
			IC_50_ (μM)	Inhibition (%)	IC_50_ (μM)	IC_50_ (μM)
IC ^a)^	Agrimoniin (AM)		13.0 ± 0.06	35.0 ± 0.41	1.6 ± 0.12	-
	Rutin (RT)		66.8 ± 0.34	31.7 ± 0.65	9.5 ± 0.75	9.0 [24] ^b)^
	Luteolin-7-*O*-glucoside (LGC)		71.5 ± 0.29	46.9 ± 0.95	8.1 ± 0.72	7.5 [26]
	Luteolin-7-*O*-glucuronide (LGN)		80.6 ± 0.38	83.3 ± 0.88	0.7 ±0.54	3.1 [27]
	Apigenin-7-*O*-glucoside (AGC)		>250	40.2 ± 0.56	4.3 ± 0.14	23.0 [28]
	Quercitrin (QU)		77.9 ± 0.27	97.4 ± 1.38	0.2 ±0.02	0.2 [29]
	Apigenin-7-*O*-glucuronide (AGN)		>250	<0	>30	-
KNC ^b)^	Catechin (CT)		106.7 ± 0.43	7.2 ± 1.02	>30	>30 [24]
	Kaempferol (KP)		91.6 ± 0.68	11.8 ± 0.81	15.2 ± 1.32	10 [24]
	Quercetin (QC)		70.4 ± 0.15	74.1 ± 0.85	3.2 ± 0.13	2.2 [24]
	Isoquercitrin (IQC)		65.9 ± 0.46	41.0 ± 1.07	5.1 ± 0.88	4.5 [24]
	Hyperin (HP)		73.3 ± 0.23	90.8 ± 0.96	4.1 ± 0.32	3.0 [24]
	Apigenin (AG)		156.3 ± 1.21	81.8 ± 1.20	3.2 ± 0.21	2.2 [24]
	Vitexin (VT)		>250	12.2 ± 0.95	>30	>30 [25]
	Astragalin (AS)		>250	53.3 ± 1.14	5.1 ± 0.89	>30 [25]
	Luteolin (LT)		88.2 ± 0.52	80.2 ± 0.90	0.6 ± 0.03	0.5 [27]
	Afzelin (AZ)		>250	86.2 ± 0.38	1.0 ± 0.27	0.3 [30]
Positive control	DPPH	l-Ascorbic acid	147.3 ± 0.43	-	-	-
	RLAR	TMG	-	119.7 ± 0.22	0.5 ± 0.05	1.0 [30]

^a)^ ICs are the compounds isolated from *A. pilosa* Ledeb; ^b)^ KNCs are the known compounds isolated from *A. pilosa* Ledeb; ^b)^ [Number] is reference number.

**Table 3:** Inhibitory effect of the constituents on the sorbitol accumulation in rat lenses and inhibition type by active compound.

Compounds	Sorbitol Content (mg)/Lens Wet Weight (g) ^a)^	Inhibition (%)	Inhibition Types (References)
Sorbitol free	No detection	-	-
Control	1.47 ± 0.04		
Quercetin ^a)^	0.21 ± 0.02	85.7 ± 8.32	Noncompetitive [31]
Agrimoniin (AM)	0.77 ± 0.02	47.6 ± 1.34	Noncompetitive
Luteolin-7-*O*-glucuronide (LGN)	0.12 ± 0.01	91.8 ± 9.01	Noncompetitive
Quercitrin (QU)	0.34 ± 0.02	76.9 ± 5.32	Uncompetitive [29]
Luteolin (LT)	0.12 ± 0.01	91.8 ± 7.91	Mixed type [30]
Afzelin (AZ)	0.10 ± 0.01	93.2 ± 8.67	Noncompetitive

^a)^ Quercetin was used as positive control; ^b)^ [Number] is reference number.

## Supplementary Materials

Supplementary materials can be found at www.mdpi.com/1422-0067/18/2/379/s1.

## Abbreviations

AR	aldose reductase
NADPH	nicotinamide adenine dinucleotide phosphate
NADH	nicotinamide adenine dinucleotide
CT	catechin
AP	*Agrimonia pilosa* Ledeb
LT	luteolin
QC	quercetin
IQC	isoquercetin
HP	hyperin
AG	apigenin
VT	vitexin
KP	kaempferol
AS	astragalin
AZ	afzelin
RLAR	rat lens aldose reductase
APE	*Agrimonia pilosa* 50% methanol (MeOH) extract
RHAR	recombinant human aldose reductase
ICs	isolated compounds isolated from *Agrimonia pilosa*
KNCs	known compounds isolated from *Agrimonia pilosa*
TMG	tetramethylene glutaric acid
PAR	peak area reduction
CH_2_Cl_2_	methylene chloride
EtOAc	ethyl acetate
*n*-BuOH	*n*-butanol
DPPH	1,1-diphenyl-2-picrylhydrazyl
