# Supplementary material for: Phytochemical Analysis of Agrimonia pilosa Ledeb, Its Antioxidant Activity and Aldose Reductase Inhibitory Potential
Source: Int J Mol Sci. 2017 Feb 10;18(2):379. doi: 10.3390/ijms18020379 (PMC5343914; doi:10.3390/ijms18020379)
Supplement: Supplementary file 1 [file ijms-18-00379-s001.pdf]

Table S1. ^{1}H and ^{13}C NMR chemical shifts of the compound isolated from *A. pilosa* Ledeb.

Agrimoniin (**1**). White amorphous powder, [α]D +162° (*c* = 0.50, MeOH); m.p. 190°C, FAB-MS *m/z* 1893 $[M+Na]^+$; ^{1}H-NMR (400 MHz, CD3OD, δH) δ 7.35 (1H, d, *J* = 2.1 Hz), 7.21 (1H, s), 6.79 (1H, d, *J* = 2.1 Hz) [dehydrodigalloyl group]; 6.63 (1H, s), 6.59 (1H, s), 6.51 (2H, s), 6.48 (1H, s), 6.47 (1H, s), 6.46 (1H, s), 6.40 (1H, s) [4 × HHDP group]; 6.61 (1H, d, *J* = 1.6 Hz) and 6.32 (1H, d, *J* = 1.7 Hz) [glucose anomeric protons], 5.60–5.40 (2H, m) [glucose H3 and H3′], 5.38–5.01 (4H, m) [glucose H2, H2′ and H4, H4′], 4.59 (2H, dd, *J* = 10.5, 6.3 Hz) and 4.39 (2H, dd, *J* = 10.5, 6.3 Hz) [glucose H5 and H5′], 3.78 (2H, d, *J* = 13.5 Hz) and 3.63 (2H, d, *J* = 13.5 Hz) [glucose H6 and H6′]. Data with agreement with Olennikov et al. [17].

Rutin (**2**). Yellow amorphous powder, [α]D -33.5° (*c* = 0.20, MeOH); m.p. 190°C; EI-MS *m/z* 611 $[M]^+$; ^{1}H-NMR (400 MHz, CD3OD, δH) 7.56 (1H, dd, *J* = 9.0, 2.1 Hz, H-6'), 7.55 (1H, d, *J* = 2.1 Hz, H-2'), 6.85 (1H, d, *J* = 8.4 Hz, H-5'), 6.40 (1H, d, *J* = 2.0 Hz, H-8), 6.11 (1H, d, *J* = 2.2 Hz, H-6), 5.35 (1H, d, *J* = 7.8 Hz, H-1''), 5.12 (1H, d, *J* = 1.6 Hz, H-1''') 3.83–3.35 (10H, m, H-2''-6ab'' and 2'''-5'''), 1.13 (3H, d, *J* = 6.2 Hz, H-6'''), ^{13}C-NMR (100 MHz, CD3OD, δc) δ 178.2 (C-4), 164.3 (C-7), 161.5 (C-5), 157.1 (C-9), 156.7(C-2), 148.7 (C-4′), 145.6 (C-3'), 134.1 (C-3), 121.9 (C-1'), 122.0 (C-6'), 116.5(C-5'), 116.1(C-2'), 104.2 (C-10), 101.4 (C-1''), 101.0 (C-1'''), 98.9 (C-6), 93.9 (C-8), 76.7 (C-3''), 76.7 (C-5''), 74.9 (C-2''), 72.7 (C-4''), 71.4 (C-4'''), 71.2 (C-3'''), 70.8 (C-2'''), 68.5 (C-5'''), 67.3 (C-6''), 18.0 (C-6''').

Luteolin-7-*O*-glucoside (**3**). White amorphous powder; [α]D +39.5° (*c* = 0.20, MeOH); m.p. 249°C; EI-MS *m/z* 449 $[M]^+$; ^{1}H-NMR (400 MHz, CD3OD, δH) 7.41 (1H, s, H-2'), 7.40 (1H, d, *J* = 8.1 Hz, H-6'), 6.82 (1H, d, *J* = 8.10 Hz, H-5'), 6.80 (d, *J* = 2.0 Hz, H-8), 6.75 (1H, s, H-3), 6.41 (1H, d, *J* = 2.0 Hz, H-6), 5.01 (1H, d, *J* = 7.3 Hz, H-1''), 3.73-3.21 (6H, m, H-2'', 3 '', 4'', 5'' and 6ab''), ^{13}C-NMR (100 MHz, CD3OD, δc) δ 182.4 (C-4), 165.1 (C-7), 163.4 (C-2), 160.9 (C-5), 157.1 (C-9), 152.9 (C-4'), 146.4 (C-3'), 120.4 (C-1'), 120.0 (C-6'), 116.5 (C-5'), 113.3 (C-2'), 105.9 (C-10), 102.9 (C-3), 100.5 (C-1''), 100.1 (C-6), 95.4 (C-8), 76.9 (C-3''), 76.8 (C-5''), 73.3 (C-2''), 70.1 (C-4''), 61.3 (C-6'').

Apigenin-7-*O*-glucuronide (**4**). Yellow amorphous powder, [α]D -40° (*c* = 0.20, MeOH); m.p. 176 °C; EI-MS *m/z* 447 $[M]^+$; ^{1}H-NMR (400 MHz, CD3OD, δH) 7.98 (2H, d, *J* = 8.7 Hz, H-2', 6'), 6.91 (2H, d, *J* = 8.7 Hz, H-3', 5'), 6.83 (1H, s, H-3), 6.81 (1H, d, *J* = 1.8 Hz, H-8), 6.42 (1H, d, *J* = 1.8 Hz, H-6), 5.12 (1H, d, *J* = 7.0 Hz, H-1''), 3.90 (1H, d, *J* = 9.71 Hz, H-5''), 3.50-3.29 (3H, m, H-2'', 3'' and 4''), ^{13}C-NMR (100 MHz, CD3OD, δc) δ 181.9 (C-4), 172.6 (C-6''), 165.1 (C-2), 163.1 (C-7), 162.1 (C-5), 159.9 (C-4'), 157.6 (C-9), 129.3 (C-2',6'), 120.8 (C-1'), 116.7 (C-3', 5'), 105.9 (C-10), 102.8 (C-3), 99.5 (C-1''), 100.2 (C-6), 95.3 (C-8), 77.2 (C-3''), 74.4 (C-5''), 73.7 (C-2''), 72.3 (C-4'').

Quercitrin (**5**). Yellow amorphous powder, [α]D -141° (*c* = 0.40, MeOH); m.p. 183°C; EI-MS *m/z* 449 $[M+H]^+$; ^{1}H-NMR (400 MHz, CD3OD, δH) δ 7.32 (1H, d, *J* = 1.5 Hz, H-2'), 7.29 (1H, dd, *J* = 8.5, 1.5 Hz, H-6'), 6.90 (1H, d, *J* = 8.5 Hz, H-5'), 6.40 (1H, d, *J* = 1.5 Hz, H-8), 6.22 (1H, d, *J* = 2.0 Hz, H-6), 5.23 (1H, d, *J* = 7.24 Hz, H-1''), 3.98 (1H, s, H-2''), 3.53–3.30 (3H, m, H-3'', 4'' and 5''), 0.79 (3H, d, *J* = 2.0 Hz, H-6''), ^{13}C-NMR (100 MHz, CD3OD, δc) δ 178.2 (C-4), 165.0 (C-7), 162.0 (C-5), 158.0 (C-2), 157.2 (C-9), 149.2 (C-4'), 145.9 (C-3'), 134.9 (C-3), 121.8 (C-6'), 121.5 (C-1'), 116.4 (C-5'), 116.2 (C-2'), 104.8 (C-10), 102.6 (C-1''), 99.4 (C-6), 94.2 (C-8), 71.9 (C-4''), 71.3 (C-2''), 71.1 (C-3''), 70.8 (C-5''), 18.2 (C-6'').

Apigenin-7-*O*-glucoside (**6**). Yellow amorphous powder; [α]D -64° (*c* = 0.40, MeOH); m.p. 204°C; EI-MS *m/z* 433 $[M+H]^+$; ^{1}H-NMR (400 MHz, CD3OD, δH) δ 7.95 (2H, d, *J* = 8.5 Hz, H-2', 6'), 6.91 (2H, d, *J* = 8.5 Hz, H-3', 5'), 6.88 (1H, s, H-8), 6.83 (1H, s, H-3), 6.45 (1H, d, *J* = 1.7 Hz, H-6), 5.14 (1H, d, *J* = 7.60 Hz, H-1''), 3.90-3.05 (6H, m, H-2'', 3'', 4'', 5'' and 6ab''), ^{13}C-NMR (100 MHz, CD3OD, δc) δ 181.7 (C-4), 164.0 (C-2), 162.7 (C-7), 161.5 (C-5), 160.8 (C-4'), 156.6 (C-9), 128.2 (C-2', 6'), 120.7 (C-1'), 115.8 (C-3', 5'), 105.1 (C-10), 102.9 (C-3), 99.8 (C-1''), 99.3 (C-6), 94.7 (C-8), 76.3 (C-3''), 77.0 (C-5''), 73.0 (C-2''), 69.5 (C-4''), 60.8 (C-6'').

Luteolin-7-*O*-glucuronide (**7**). Yellow amorphous powder; $[\alpha]_D$ +52.4° (*c* = 0.30, MeOH) m.p. 210 °C; EI-MS *m*/*z* 461 $[M-H]^-$; ^{1}H-NMR (400 MHz, CD_3OD, δ_H) δ 7.45 (1H, dd, *J* = 8.0, 2.0 Hz, H-6'), 7.44 (1H, d, *J* = 2.0 Hz, H-2'), 6.91 (1H, d, *J* = 8.3 Hz, H-5), 6.81 (1H, d, *J* = 2.0 Hz, H-8), 6.75 (1H, s, H-3), 6.46 (1H, d, *J* = 2.2 Hz, H-6), 5.31 (1H, d, *J* = 7.32 Hz, H-1''), 4.10 (1H, d, *J* = 9.5 Hz, H-5''), 3.41-3.24 (3H, m, H-2'', 3'' and 4''), ^{13}C-NMR (100 MHz, CD_3OD, δ_C) δ 180.9 (C-4), 173.1 (C-6''), 165.4 (C-2), 163.2 (C-7), 160.9 (C-5), 153.1 (C-9), 153.9 (C-4'), 147.4 (C-3'), 119.8 (C-6'), 118.2 (C-1'), 116.5 (C-5'), 112.7 (C-2'), 104.9 (C-10), 101.9 (C-3), 101.1 (C-1''), 99.8 (C-6), 93.9 (C-8), 76.88 (C-3''), 73.9 (C-5''), 73.3 (C-2''), 72.1 (C-4'').
